# Non-specific uptake of ^18^F-FAPI-04 in the pancreas and its related factors: a post-hoc analysis of an ongoing prospective clinical trial

**DOI:** 10.1038/s41598-024-62005-2

**Published:** 2024-05-15

**Authors:** Yan Li, Jungang Gao, Yang Li, Xiaoyi Duan, Cong Shen

**Affiliations:** Department of PET/CT, The First Affiliated Hospital of Xi’an Jiaotong University, 277 Yanta West Road, Xi’an, 710061 Shaanxi China

**Keywords:** ^18^F-FAPI, ^18^F-FDG, PET/CT, Non-specific uptake; pancreas, Biomarkers, Diseases, Health care, Medical research

## Abstract

This study aimed to analyze the characteristics of the non-specific uptake (NSU) of ^18^F-labeled fibroblast activation protein inhibitor (^18^F-FAPI) of the pancreas and investigate the related factors. Totally, 78 patients who underwent both ^18^F-fluorodeoxyglucose (FDG) and ^18^F-FAPI PET/CT examinations were divided into normal (n = 53) and NSU (n = 25) groups. The differences in general information, medical history, laboratory indexes and uptake were compared. Receiver operating characteristic (ROC) curves were used to analyze the optimal cut-off values. The correlations between ^18^F-FAPI-SUVmax and blood cell analysis, liver function indexes, tumor markers, and inflammatory indices were analyzed. The logistic regression model was used to estimate the independent factors. Both ^18^F-FAPI (4.48 ± 0.98 vs. 2.01 ± 0.53, *t* = 11.718, *P* < 0.05) and ^18^F-FDG (2.23 ± 0.42 vs. 2.02 ± 0.44, *t* = 2.036, *P* = 0.045) showed significantly higher in NSU group. Patients in the NSU group tended to be complicated with a history of drinking (*P* = 0.034), chronic liver diseases (*P* = 0.006), and surgery of gastrectomy (*P* = 0.004). ROC analysis showed cutoff values of 3.25 and 2.05 for ^18^F-FAPI and ^18^F-FDG in identifying the NSU. Patients in the NSU group showed less platelet count, higher platelet volume, higher total bilirubin, direct or indirect bilirubin (*P* < 0.05). Platelet count, platelet crit, large platelet ratio, aspartate aminotransferase (AST), α-l-fucosidase, and total, direct or indirect bilirubin were correlated with ^18^F-FAPI-SUVmax (*P* < 0.05). AST [1.099 (1.014, 1.192), *P* = 0.021] and total bilirubin [1.137 (1.035, 1.249), *P* = 0.007] were two independent factors in the step forward logistic regression, and platelet/% [1.079 (1.004, 1.160), *P* = 0.039] and total bilirubin [1.459 (1.016, 2.095), *P* = 0.041] were two independent factors in the step backward logistic regression for the prediction of pancreatic uptake of ^18^F-FAPI. ^18^F-FAPI-PET/CT was better than ^18^F-FDG in predicting the pancreatic NSU, and NSU is related to a history of drinking, chronic liver diseases, gastrectomy, heteromorphic platelet, and impaired liver function.

## Introduction

Fibroblast activation protein (FAP), a type II transmembrane glycoprotein, is an antigenic molecule on the surface of cancer-associated fibroblasts (CAFs)^1,2^. FAP is often highly expressed in tumors of epithelial origin^3^, and FAP inhibitors (FAPIs) are small molecule compounds synthesized based on the structure of quinoline, which can bind specifically to FAP on the surface of CAFs. Thus, FAPI positron emission tomography/computed tomography (PET/CT) is commonly used in the clinical imaging of malignancies. Besides, FAP can also be found during the remodeling of the extracellular matrix, such as chronic inflammation, arthritis, fibrosis, and ischemic heart tissue after a myocardial infarction^4^. Thus, non-oncological FAPI uptake can be seen in liver fibrosis, cirrhosis, arthritis, cardiovascular disease, IgG4-related disease, and benign tumors^5^.

The pancreas, a vital digestive organ, is surrounded by a fibrous capsule from which connective tissue septa extend into the gland. FAPI is highly expressed in pancreatic cancers due to the abundant extracellular matrix and stromal cells, including CAFs. Although mesenchyme accounts for approximately 15–25% of total pancreas volume^6^, FAPI uptake was low in the normal tissues of the pancreas due to the inactivated mesenchyme fibroblast, with a maximum standardized uptake value (SUVmax) of 1.04–3.0, even lower than the FDG uptake^7–9^.

Except for the oncological uptake, FAPI accumulation has also been confirmed in acute pancreatitis (average SUVmax: 7.5 ± 3.5, n = 8)^10^ and IgG4-related pancreatitis (average SUVmax: 15.2 ± 9.0, n = 19)^11^. Zhang^12^ has reported 7 cases of focally elevated uptake of ^68^Ga-FAPI-04 in the pancreas, and lesions were identified, including pancreatic pseudocysts, sites of prior pancreatitis, and IgG 4-related disease. However, in our clinical observation, we noticed that some cases without pancreatic cancer and acute or IgG4-related pancreatitis also showed an accumulation of ^18^F-FAPI in the pancreas. Rare studies have mentioned this non-specific uptake (NSU) of FAPI in the pancreas. Thus, this study aimed to analyze the characteristics of the NSU of ^18^F-FAPI of the pancreas and investigate the potential independent factors.

## Materials and Methods

### Materials

This was a post-hoc analysis of a single-center, ongoing prospective clinical trial conducted at the First Affiliated Hospital of Xi’an Jiaotong University (approval number: XJTU1AF-CRF-2022-021). The study protocol was approved by the Ethics Committee of Xi’an Jiaotong University (XJTU1AF2022LSL-021). This study was registered at ClinicalTrials Gov (number NCT05788874). All participants signed an informed consent form following the national regulations of the Declaration of Helsinki and Good Clinical Practice.

**Figure 1:**
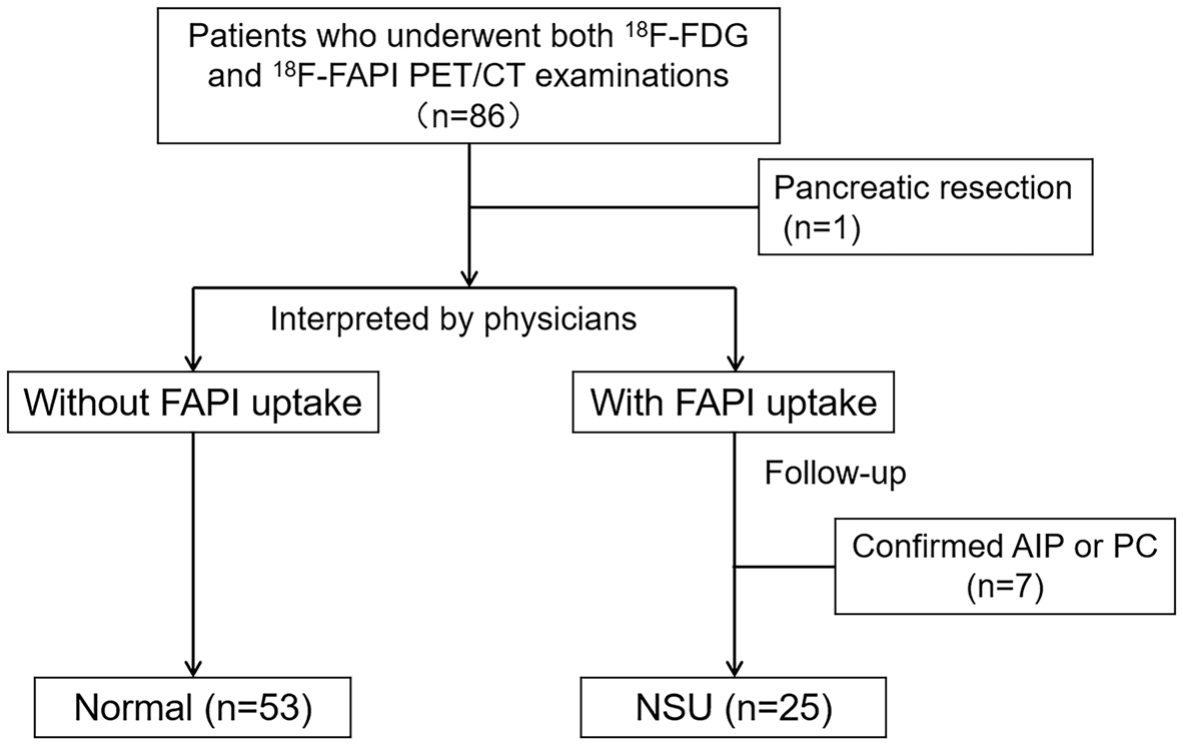
Study workflow. *FAPI* fibroblast activation protein inhibitor, *FDG* fluorodeoxyglucose, *AIP* autoimmune pancreatitis, *PET/CT* positron emission tomography/computed tomography, *NSU* non-specific uptake, *PC* pancreatic cancer.

This retrospective study included 86 patients who underwent both ^18^F-FDG and ^18^F-FAPI PET/CT examinations at an interval of 1–43 days. One patient with a history of pancreatic resection was excluded, and all patients were followed up for the final diagnosis. 4 cases of autoimmune pancreatitis (AIP) and 3 cases of pancreatic cancer (PC) confirmed by imaging manifestations, or histopathological evidence, were excluded. None of the women included in this study were pregnant. The study flow is displayed in Fig. 1.

### Methods

#### PET/CT image acquisition

##### Preparation of ^18^F-FDG and ^18^F-FAPI-04

^18^F-FDG and ^18^F-FAPI-04 were synthesized using a GE MINItrace cyclotron and Tracerlab FX-FN multifunctional synthesizer. The synthetic precursor kits, FDG and FAPI, were purchased from ABX (Germany) and Jiangyuan (China). The radio-chemical purity of the synthesized drugs exceeded 95%. Further physical and biological quality controls were performed in compliance with current pharmacopeias.

##### PET/CT imaging

All patients underwent PET/CT on the same scanner (Gemini TF PET/CT, Philips, Netherlands). For ^18^F-FDG PET/CT scans, the patients fasted for ≥ 6 h, and the blood glucose levels were ≤ 12 mmol/L before the injection (370 MBq/kg). There was no special preparation for patients on the day of ^18^F-FAPI PET/CT scanning, and ^18^F-FAPI PET/CT scan was initiated 60 min after injection (370 MBq/kg).

Images encompassing the head to the mid-thigh were obtained for both scans. PET scans (1.5 min/bed position, 6–10 bed/per person) were acquired with an interval of 68.5 ± 12.1 min (range, 47 ~ 90 min) in three-dimensional mode after the injection. CT scans were performed (tube voltage 120 kV, tube current 50–100 mAs, layer thickness, 5 mm; 512 × 512 matrix) for attenuation correction and anatomical reference. The images were then reconstructed using an iterative method and time of flight, and the image data were transferred to an Extended Brilliance Workstation for image interpretation.

#### PET/CT image evaluation

The ^18^F-FDG and ^18^F-FAPI PET/CT images were interpreted by two > 10 years of experienced PET/CT physicians (Y.L.) and (C.S.), and a consensus was performed. Any disagreements were resolved by a more experienced PET/CT physician. The NSU group was defined as the increased radioactivity uptake of FAPI in the pancreas compared to the background, without confirming pancreatic cancer, acute or IgG4-related pancreatitis by other imaging methods, serology, or pathological results (if available).

For the normal group, the criteria were: (1) without ^18^F-FAPI uptake compared with the background; (2) without elevated serum tumor biomarkers or pancreatitis biomarkers; (3) without a history of pancreatic disease; or (4) without pancreatic-related symptoms or body signs.

The same nuclear medicine physician (C.S.) measured the pancreas' SUVmax values using circular regions of interest (ROI) placed on axial slices. The CT image features of NSU patients, including pancreatic swelling, calcification, and irregular dilatation of the pancreatic ducts, were also evaluated.

#### Clinical indexes

General clinical indices, including sex (male/female), age (years), primary diagnosis (specific name), height (cm), weight (kg), and BMI (kg*m^−2^), were recorded. The medical history, including smoking (no/yes), drinking (no/yes), hypertension (no/yes), diabetes (no/yes), chronic liver diseases (no/yes), active tumor (no/yes), chemotherapy (no/yes), gastrectomy (no/yes), intestine enterectomy (no/yes) and intrahepatic metastasis (no/yes) were recorded.

#### Laboratory test indexes

Laboratory tests at an interval of 3 days with the ^18^F-FAPI-PET/CT scan were recorded (if available), including the following parameters:

Blood cell indicators: red blood cell count (10^12^*L^−1^), hemoglobin (g*L^−1^), hematocrit (%), mean erythrocyte volume (fL), average hemoglobin (pg), average hemoglobin concentration (g*L^−1^), erythrocyte width-CV (%), erythrocyte width SD (fL), platelet count (10^9^*L^−1^), platelet distribution width (fL), mean platelet volume (fL), large platelet ratio (%), platelet accumulation (%), white blood cell count (10^9^*L^−1^), lymphocyte count (10^9^*L^−1^), monocyte count (10^9^*L^−1^), neutrophil count (10^9^*L^−1^), eosinophilic cell count (10^9^*L^−1^), basophilic cell count (10^9^*L^−1^).

Liver function indicators: aspartate aminotransferase (AST) (U*L^−1^), alanine transaminase (ALT) (U*L^−1^), AST/ALT, alkaline phosphatase (U*L^−1^), γ-glutamyl transpeptidase (U*L^−1^), total bilirubin (TBIL) (μmol*L^−1^), direct bilirubin (DBIL) (μmol*L^−1^), indirect bilirubin (IDBIL) (μmol*L^−1^), total protein (g*L^−1^), albumin (g*L^−1^), globulin (g*L^−1^), albumin-globulin ratio (%), total bile acids (μmol*L^−1^), blood glucose (mmol*L^−1^), and α-L-fucosidase (U*L^−1^).

Tumor markers: squamous cell carcinoma (SCC) (ng*mL^−1^), carcinoembryonic antigen (CEA) (ng*mL^−1^), alpha-fetoprotein (AFP) (ng*mL^−1^), carbohydrate antigen 125 (CA-125) (U*mL^−1^), CA-199 (U*mL^−1^), CA-724 (U*mL^−1^), cytokeratin fragment 19 (CYFRA-19) (ng*mL^−1^), and neuron-specific enolase (NSE) (ng*mL^−1^).

Inflammatory indices: C-reactive protein (CRP) (mg*L^−1^), interleukin 6 (IL-6) (pg*mL^−1^), IL-10 (pg*mL^−1^), IL-17 (pg*mL^−1^).

#### Statistical analysis

IBM SPSS Statistics for Windows, version 25.0 (IBM Corp., Armonk, NY, USA) was used for the statistical analyses. Continuous variables with normal distribution and variance uniformity were represented as means ± standard deviation and analyzed by independent-sample *t*-tests, and continuous variables without normal distribution were expressed as median [25%, 75%] and were tested using the Mann–Whitney U test. Categorical variables were defined as several cases and analyzed by χ^2^ or Fisher exact tests. *Kappa* test was used for consistency evaluation between the two readers.

Receiver operating characteristic (ROC) curves were used to analyze the optimal cut-off value of SUVmax for predicting NSU of the pancreas [determined by Youden index = sensitivity + specificity − 1]. The DeLong test was used to compare the performance between the ^18^F-FAPI and ^18^F-FDG using Medcalc (v22.009, MedCalc Software Ltd, Belgium).

The correlations between FAPI-SUVmax and blood cell analyzed indicators, liver function indicators, tumor markers, and inflammatory indices were analyzed by Pearson correlation (only continuous variables with normal distribution and variance uniformity) or Spearman correlation. The logistic regression model was used to estimate the factors related to pancreatic NSU. Statistical significance was set at *P* < 0.05.

## Results

### Clinical characteristics

Seventy-eight patients (45 males, 33 females) were analyzed with a median age of 60 years old (25% ~ 75%, 52 ~ 68 years old). The primary malignancy or the primary diagnosis was shown in Supplementary Table 1.

Among the 78 patients included, 25 (32.05%) showed NSU in the pancreas with a pattern of diffused uptake. For the 25 cases in the NSU group, the agreement rate of the two readers was 84.00% (21/25), and 4 cases (with the SUVmax of 2.57, 3.33, 3.41 and 3.41, respectively) were judged by the 3rd reader. For the 53 cases in the normal group, the agreement rate of the two readers was 98.11% (52/53), and 1 cases (with the SUVmax of 3.17) were judged by the 3rd reader. The Kappa of the two readers was 0.848 (*P* < 0.001). None of the patients in the NSU patients showed pancreatic swelling and calcification, and only one patient (4%) showed irregular dilatation of the pancreatic ducts. The tracer uptake of ^18^F-FAPI showed significantly higher in the NSU group than in the normal group (4.48 ± 0.98 vs. 2.01 ± 0.53, *t* = 11.718, *P* < 0.001). Meantime, although with the slight difference of SUVmax, the tracer uptake of FDG also showed significantly higher in the NSU group than that of in the normal group (2.23 ± 0.42 vs. 2.02 ± 0.44, *t* = 2.036, *P* = 0.045), see as Table 1.

**Table 1:** Clinical characteristics of the patients in two groups.

Items	Normal (n = 53)	NSU (n = 25)	T/Z/χ^2^	P
Genaral information				
Age/year	60 (51, 68)	58.40 ± 10.80	− 0.182^a^	0.940
Sex (male/female)	29/24	16/9	0.600^b^	0.439
Height/cm	165.36 ± 7.608	166.16 ± 8.035	− 0.426	0.671
Weight/kg	60 (55, 69)	60.21 ± 12.38	− 0.182^a^	0.855
BMI/kg*m^−2^	22.16 ± 3.18	21.76 ± 3.71	0.482	0.631
Medical history				
Smoking (no/yes)	45/8	19/6	0.915^b^	0.339
Drinking (no/yes)	52/1	21/4	NA	0.034
Hypertension (no/yes)	41/12	18/7	0.265^b^	0.607
Diabetes (no/yes)	46/7	21/4	0.109^b^	0.741
Chronic liver diseases (no/yes)	50/3	18/7	7.585^b^	0.006
Active tumor (no/yes)	10/43	5/20	0.014^b^	0.906
Chemotherapy (no/yes)	35/18	15/10	0.269^b^	0.604
Gastrectomy (no/yes)	48/5	16/9	NA	0.004
Intestine enterectomy (no/yes)	49/4	22/3	NA	0.674
Intrahepatic metastasis (no/yes)	44/9	22/3	NA	0.742
Uptake of tracers				
SUVmax_FAPI	2.01 ± 0.53	4.48 ± 0.98	11.718	< 0.001
SUVmax_FDG	2.02 ± 0.44	2.23 ± 0.42	2.036	0.045
Interval time of two scans (day)	2 (1, 4)	2 (1, 6)	− 0.734	0.463

*NSU* non-specific uptake, *BMI* body mass index, *FAPI* fibroblast activation protein, *FDG* fluoro-deoxyglucose.

^a^Z test. ^b^*x*^*2*^* test*; NA: Fisher test.

**Figure 2:**
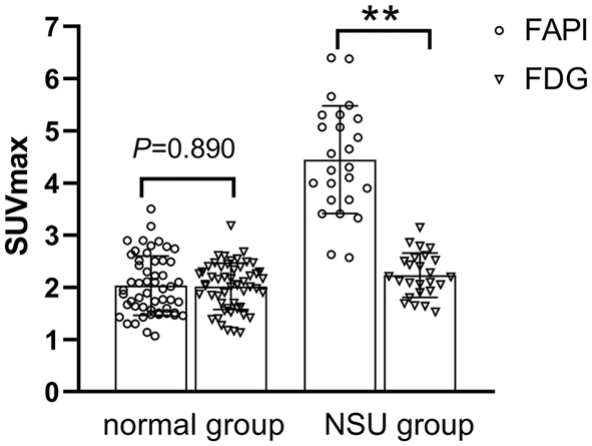
Comparison of SUVmax values. FAPI, fibroblast activation protein inhibitor; FDG, fluorodeoxyglucose; NSU, non-specific uptake; **P < 0.01.

The tracer uptake in the normal group between the ^18^F-FAPI and ^18^F-FDG showed no difference (*P* = 0.890), while the tracer uptake in the NSU group between ^18^F-FAPI and ^18^F-FDG showed a significant difference (*P* < 0.001), see as Fig. 2.

**Figure 3:**
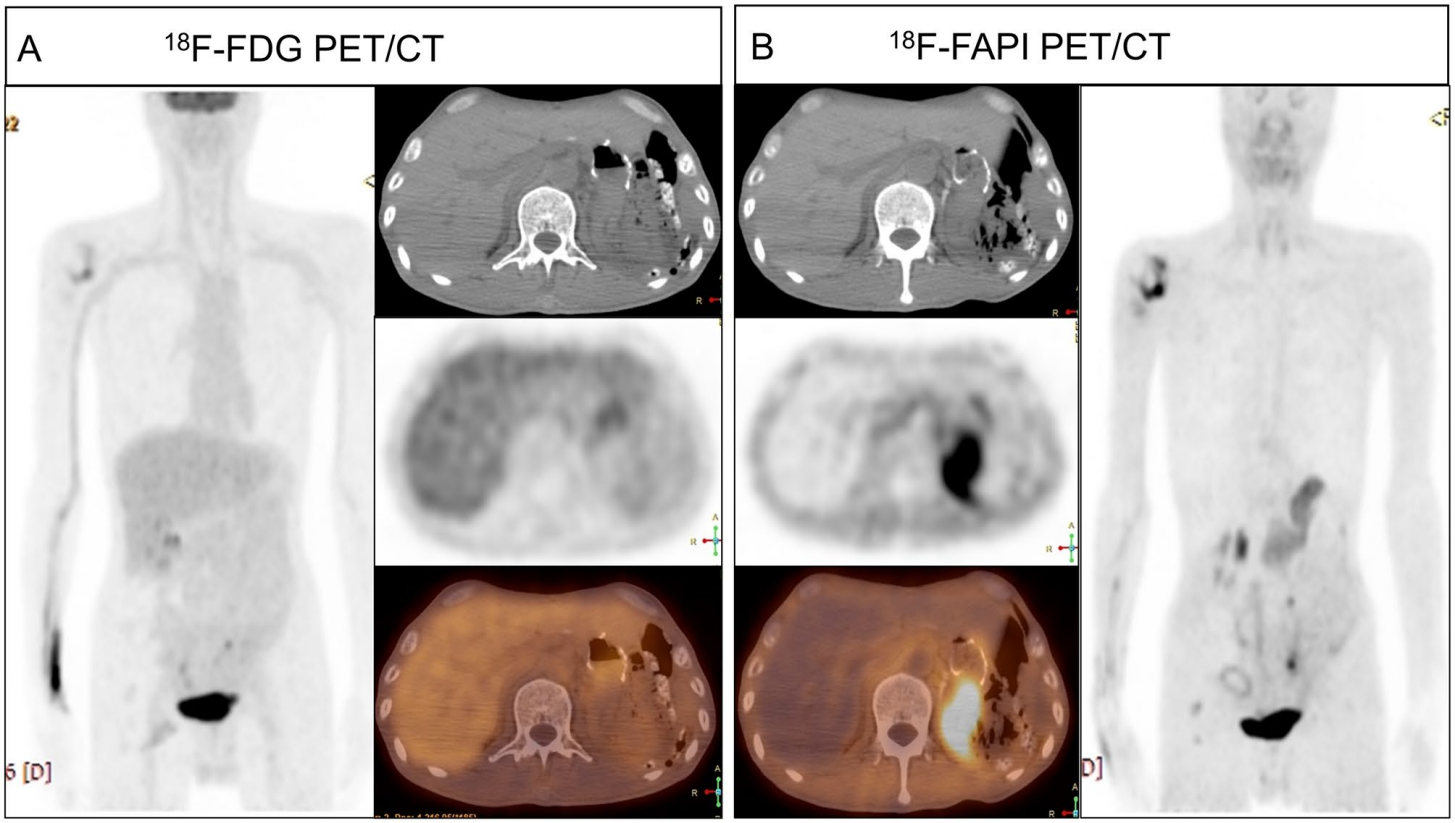
NSU in the ^18^F-FAPI after the surgery of gastrectomy and negative in the ^18^F-FDG PET/CT. A male patient, 55 years old, after surgery of gastrectomy for gastric cancer. ^18^F-FDG PET/CT (**A**) showed no FDG concentration in the pancreas, and ^18^F-FAPI PET/CT imaging (**B**) showed a high concentration of diffuse FAPI in the pancreas, with an SUVmax of 5.3. *FAPI* fibroblast activation protein, *FDG* fluorodeoxyglucose, *PET/CT* positron emission tomography/computed tomography. *SUVmax* maximum standardized uptake value.

**Figure 4:**
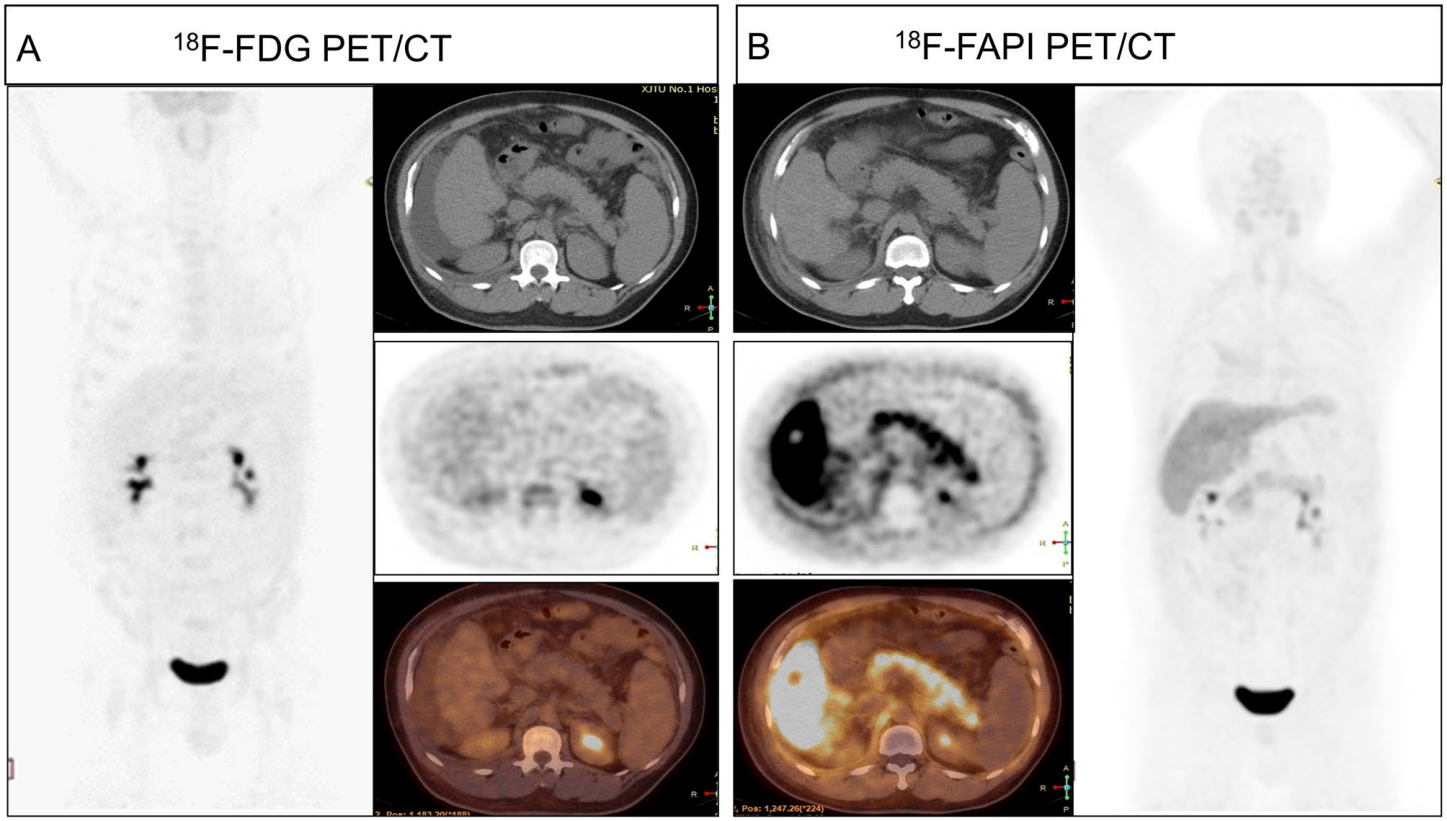
NSU in the ^18^F-FAPI of the cirrhosis of the liver and negative in the ^18^F-FDG PET/CT. A male patient, 42 years of age, with chronic cirrhosis of the liver. ^18^F-FDG PET/CT (**A**) showed no FDG concentration in the pancreas, and ^18^F-FAPI PET/CT imaging (**B**) showed a high concentration in the pancreas, with an SUVmax of 6.3. *FAPI* fibroblast activation protein, *FDG* fluorodeoxyglucose, *PET/CT* positron emission tomography/computed tomography, *SUVmax* maximum standardized uptake value.

In the Chi-Square test, patients in the NSU group weretended to complicate with a history of drinking (*P* = 0.034), chronic liver diseases (*P* = 0.006), and surgery of gastrectomy (*P* = 0.004), see Table 1, Figs. 3 and 4.

### ROC analysis of SUVmax value of two tracers

**Figure 5:**
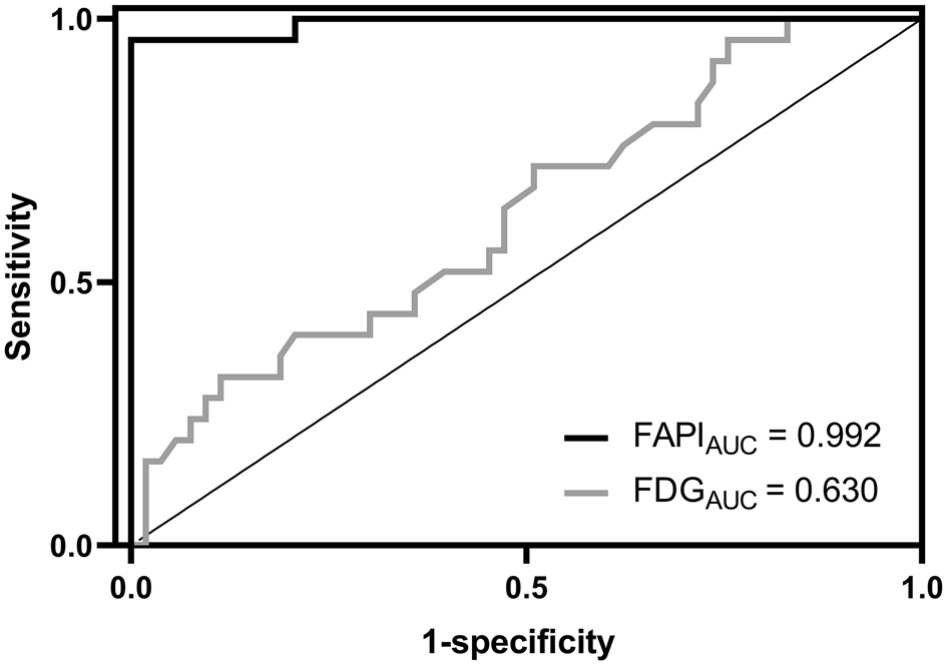
ROC analysis of SUVmax for ^18^F-FAPI and ^18^F-FDG in predicting pancreatic NSU.

ROC analysis showed that the cutoff value for FAPI-SUVmax to identify the NSU from normal was 3.25, with an area under the curve (AUC), sensitivity, and specificity of 0.992, 0.960, and 1.000, respectively, see Fig. 5. The cutoff value for FDG-SUVmax to identify the NSU from normal was 2.05, with an AUC an area under the curve (AUC), sensitivity, and specificity of 0.630, 0.720, and 0.509, respectively, see Table 2. The Delong test showed that the two ROC curves differed significantly (Z = 5.348, *P* < 0.001).

**Table 2:** SUVmax cutoff values.

Variable	AUC (95%CI)	Sensitivity	Specificity	Cutoff value	Youden index
SUVmax_FAPI	0.992 (0.975 ~ 1.000)	0.960	1.000	3.25	0.960
SUVmax_FDG	0.630 (0.499 ~ 0.761)	0.720	0.509	2.05	0.229

*FAPI* fibroblast activation protein inhibitor, *FDG* fluorodeoxyglucose, *NSU* non-specific uptake.

### Comparisons of blood cell analysis, liver function indicators, tumor biomarkers, and inflammatory indices between two groups

Patients in the NSU group showed a lower level of platelet count (NSU *vs.* Normal, 182.26 ± 76.70 *vs.* 229.30 ± 74.17,* t* = 2.490, *P* = 0.015), higher level of mean platelet volume (NSU *vs.* Normal, 10.93 ± 1.34 *vs.* 10.26 ± 1.23,* t* = − 2.108, *P* = 0.039), and large platelet ratio (NSU *vs.* Normal, 32.65 ± 10.05 *vs.* 27.47 ± 8.40,* t* = − 2.298, *P* = 0.025) for the complete blood cells analysis, and patients in the NSU group showed a higher level of total bilirubin (NSU *vs.* Normal, 21.37 ± 15.84 *vs.* 9.55[6.62, 13.05],* Z* = − 3.848, *P* < 0.001), direct bilirubin (NSU *vs.* Normal, 7.41 ± 6.96 *vs.* 2.75(1.87, 3.90),* Z* = − 3.290, *P* = 0.001), and indirect bilirubin (NSU *vs.* Normal, 13.95 ± 9.67 *vs.* 7.97 ± 4.56,* t* = − 2.725, *P* = 0.011) in the serum, see as Table 3. The tumor biomarkers and the inflammatory indices didn’t statistically differ in the two groups (*P* > 0.05), see Table 4.

**Table 3:** Comparisons blood cells and liver function indexes in two groups.

Items	Normal (n = 53)	NSU (n = 25)	T/Z	P
Blood analysis				
Red cell count/10^12^*L^−1^	4.07 ± 0.68	4.11 ± 0.85	− 0.212	0.833
Hemoglobin/ g*L^−1^	121.80 ± 19.96	120.08 ± 14.389	0.376	0.708
Hematocrit/%	37.79 ± 5.72	37.26 ± 4.97	0.389	0.698
Mean erythrocyte volume/fL	93.38 ± 7.75	92.19 ± 10.00	0.563	0.575
hemoglobin/pg	30.60 (29.05, 31.60)	29.88 ± 3.96	− 0.393^a^	0.695
Hemoglobin concentration/g*L^−1^	323.50(316.75,327.25)	324.33 ± 14.77	− 0.732^a^	0.470
Erythrocyte width-CV/%	13.20 (12.75, 15.47)	13.90 (13.20, 16.00)	− 1.854^a^	0.064
Erythrocyte width SD/fL	46.55(42.15, 50.90)	47.00(42.20, 54.40)	− 0.618^a^	0.537
Platelet count/10^9^*L^−1^	229.30 ± 74.17	182.26 ± 76.70	2.490	0.015
Platelet distribution width/fL	15.70(11.12, 16.30)	15.90 (12.30, 16.80)	− 1.230^a^	0.219
Mean platelet volume/fL	10.26 ± 1.23	10.93 ± 1.34	− 2.108	0.039
Large platelet ratio/%	27.47 ± 8.40	32.65 ± 10.05	− 2.298	0.025
Platelet accumulation/%	0.23 ± 0.07	0.19 ± 0.08	1.886	0.063
White cell count/10^9^*L^−1^	5.52 ± 1.83	5.49 ± 2.25	0.071	0.944
Lymphocyte count/10^9^*L^−1^	1.29 ± 0.55	1.26 ± 0.69	0.183	0.856
Monocyte count/10^9^*L^−1^	0.40 ± 0.15	0.41(0.29, 0.56)	− 0.202^a^	0.840
Neutrophil count/10^9^*L^−1^	3.67 ± 1.49	3.59 ± 2.05	0.182	0.856
Eosinophilic cell count/10^9^*L^−1^	0.09(0.05, 0.18)	0.08(0.04,0.11)	− 1.371^a^	0.170
Basophilic cell count/10^9^*L^−1^	0.02(0.02, 0.03)	0.02(0.01, 0.03)	− 0.344^a^	0.731
Liver function test				
AST/U*L^−1^	21.95 ± 6.21	21.0(18.5, 37.5)	− 1.675^a^	0.094
ALT/U*L^−1^	21.29 ± 9.46	25.33 ± 16.86	− 1.091	0.284
AST/ALT	1.21 ± 0.53	1.43 ± 0.69	− 1.562	0.123
Alkaline phosphatase/U*L^−1^	82.5(66.5, 94.0)	107.71 ± 52.18	− 1.117^a^	0.264
γ-glutamyl transpeptidase /U*L^−1^	25.5(17.2, 41.0)	27.0(18.0, 62.7)	− 0.167^a^	0.867
TBIL/μmol*L^−1^	9.55(6.62, 13.05)	21.37 ± 15.84	− 3.848^a^	< 0.001
DBIL/μmol*L^−1^	2.75(1.87, 3.90)	7.41 ± 6.96	− 3.290^a^	0.001
IDBIL/μmol*L^−1^	7.97 ± 4.56	13.95 ± 9.67	− 2.725	0.011
Total protein/g*L^−1^	67.5(64.37, 74.00)	67.81 ± 7.01	− 0.141^a^	0.888
Albumin /g*L^−1^	39.25 ± 5.19	39.01 ± 5.32	0.323	0.748
Globulin/g*L^−1^	27.65(25.02, 29.97)	28.80 ± 4.15	− 0.405^a^	0.685
Albumin-globulin ratio/%	1.50(1.30, 1.60)	1.38 ± 0.23	− 0.503^a^	0.615
Total bile acids/μmol*L^−1^	3.15(1.70, 3.90)	2.70(1.37, 9.17)	− 0.266^a^	0.790
Blood glucose/mmol*L^−1^	4.93 (4.49, 5.36)	4.73(4.37, 6.72)	0.705^a^	0.702

4 patients in normal group and 1 patient in NSU group were missed in the blood cell analysis; 4 patients in normal group and 2 patients in NSU group were missed in the liver function analysis; 34 patients in normal group and 10 patients in NSU group were included in the total bile acids comparison; 48 patients in normal group and 21 patients in NSU group were included in the blood glucose comparison;

*NSU* non-specific uptake, *FAPI* fibroblast activation protein, *FDG* fluoro-deoxyglucose, *CV* coefficients of variation, *SD* standard deviation, *AST* aspartate aminotransferase, *ALT *alanine transaminase, *TBIL* total bilirubin, *DBIL* direct bilirubin, *IDBIL* indirect bilirubin.

^a^Z test.

**Table 4:** Comparisons tumor biomarkers and inflammatory indices indexes in two groups.

Items	Normal (n = 53)	NSU (n = 25)	Z	P
Tumor biomarkers				
SCC/ ng*mL^−1^	0.66(0.89, 1.36)	0.74(0.86, 0.96)	− 0.213	0.832
CEA/ ng*mL^−1^	1.85(3.86, 13.45)	2.75(3.04, 5.61)	− 0.831	0.406
AFP/ ng*mL^−1^	2.375(3.02, 4.65)	3.5(4.44, 7.21)	− 0.900	0.368
CA-125/U*mL^−1^	9.45(24.30, 76.6)	13.8(60.60, 502)	− 1.597	0.110
CA-199/U*mL^−1^	10.65(18.90, 32.5)	7.6(64.40, 94.60)	− 1.048	0.295
CA-724/U*mL^−1^	1.135(1.84, 12.68)	1.91(3.42, 6.85)	− 0.597	0.551
CYFRA-19/ ng*mL^−1^	1.86(2.70, 4.03)	2.88(3.32, 4.74)	− 1.364	0.173
NSE/ ng*mL^−1^	12.335(14.87, 16.71)	13.63(15.58, 22.23)	− 0.304	0.761
Inflammatory indices				
CRP/ mg*L^−1^	6.58(1.52, 40.50)	2.06 (0.86, 13.70)	0.606	0.856
IL-6/pg*mL^−1^	2.53(1.75, 7.72)	6.92 (1.21, 11.67)	0.967	0.307
IL-10/pg*mL^−1^	1.93(1.05, 2.49)	2.25(1.81, 3.57)	0.980	0.292
IL-17/pg*mL^−1^	3.27(3.27, 4.49)	3.35(3.27, 12.42)	0.580	0.890

31, 40, 30, 34, 34, 32, 32, 31, 19, 17, 15, 15 patients in the normal group and 10, 20, 9, 10, 19, 14, 11, 10, 11, 11, 10, 9 in the NSU group were included for the comparisons of SCC, AFP, CA-125, CA-199, CA-724, CYFRA-19, NSE, CRP, IL-6, IL-10, IL-17, respectively.

*NSU* non-specific uptake, *CA* carbohydrate antigen, *AFP* alpha-fetoprotein, *CEA* carcinoembryonic antigen, *SCC* squamous cell carcinoma antigen, *NSE* neuron-specific enolase, *CRP* C-reactive protein, *IL* interleukin, *CYFRA-19* cytokeratin fragment 19.

### Correlation analysis of laboratory indicators with the SUVmax value of ^18^F-FAPI

**Figure 6:**
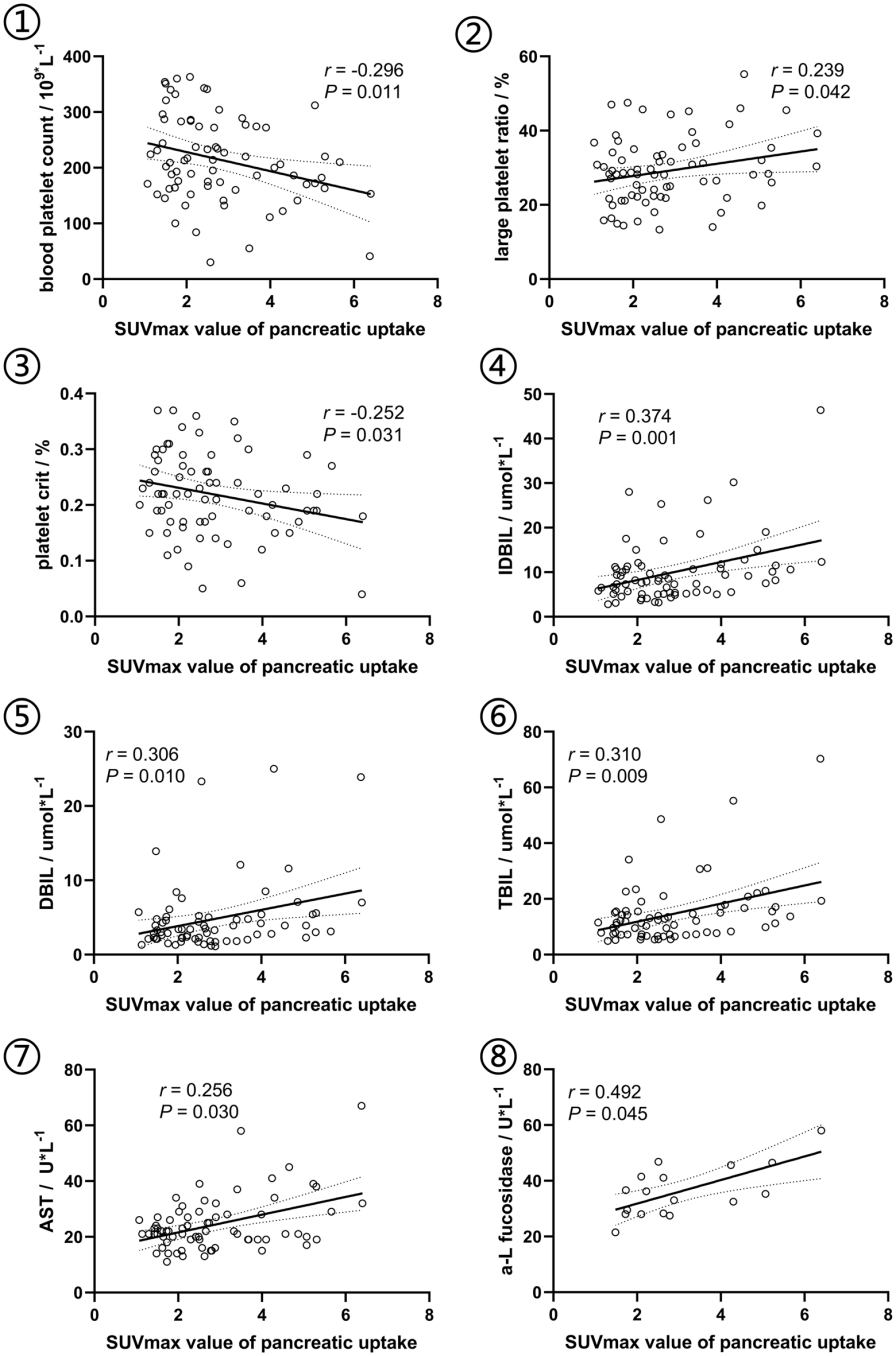
Scatter diagrams of the correlations of laboratory indicators and ^18^F-FAPI uptake. *AST* aspartate aminotransferase, *TBIL* total bilirubin, *DBIL* direct bilirubin, *IDBIL* indirect bilirubin, *SUVmax* maximum standardized uptake value.

In the correlation analysis, blood platelet count and platelet crit showed significantly negative correlation with SUVmax (*r* = − 0.296, *P* = 0.011; *r* = 0.252, *P* = 0.031, respectively), and large platelet ratio, TBIL, DBIL, IDBIL, AST, and α-K-fucosidase showed positive correlation with SUVmax (*r* ranged 0.239 ~ 0.472, all *P* value < 0.05), see as Fig. 6. Other correlations without statistical significance were shown in the Supplementary Table.

### Logistic regression of all the factors related to pancreatic uptake of ^18^F-FAPI PET/CT images

In the logistic regression, AST/U*L^−1^ [OR (95% CI), 1.099 (1.014, 1.192), *P* = 0.021] and TBIL/μmol*L^−1^[OR (95%CI), 1.137 (1.035, 1.249), *P* = 0.007] were two independent factors for the pancreatic uptake of ^18^F-FAPI with the step forward for the selection of the factors. The ratio of large platelet/% [OR (95% CI), 1.079 (1.004, 1.160), *P* = 0.039] and TBIL/μmol*L^−1^ [OR (95% CI), 1.459 (1.016, 2.095), *P* = 0.041] were two independent factors for the pancreatic uptake of ^18^F-FAPI with the step backward for the selection of the factors, see as Table 5.

**Table 5:** Logistic regression of the all the factors related with pancreatic uptake of ^18^F-FAPI PET/CT images.

	Single factor Logistic regression		multivariate logistic regression (forward)		multivariate logistic regression (backward)	
	OR (95% CI)	P	OR (95% CI)	P	OR (95% CI)	P
Drinking (no/yes)	9.905 (1.045, 93.893)	0.046				
Chronic liver diseases (no/yes)	6.481 (1.511, 27.794)	0.012				
Gastrectomy (no/yes)	16.421(1.854, 145.44)	0.012				
Platelet count/10^9^*L^−1^	0.991 (0.984, 0.999)	0.020				
Mean platelet volume/fL	1.503 (1.007, 2.243)	0.046				
Large platelet ratio/%	1.065 (1.006, 1.128)	0.030			1.079 (1.004, 1.160)	0.039
AST/U*L^−1^	1.084 (1.018, 1.153)	0.011	1.099 (1.014, 1.192)	0.021		
TBIL/μmol*L^−1^	1.135 (1.041, 1.237)	0.004	1.137 (1.035, 1.249)	0.007	1.459 (1.016, 2.095)	0.041
DBIL/μmol*L^−1^	1.289 (1.053, 1.578)	0.014				
IDBIL/μmol*L^−1^	1.159 (1.043, 1.288)	0.006				
α-L-fucosidase/U*L^−1^	1.166 (0.990, 1.373)	0.066				

*AST* aspartate aminotransferase, *ALT* alanine transaminase, *TBIL* total bilirubin, *DBIL* direct bilirubin, *IDBIL* indirect bilirubin, *OR* odds ratio, *CI* confidence interval.

## Discussion

This study tried to analyze the characteristics of the NSU uptake of ^18^F-FAPI in the pancreas by comparing it with the ^18^F-FDG head-to head. In the previous studies, non-oncological FAPI uptake was mainly explained as the pancreatic pseudocysts, sites of prior pancreatitis, and IgG 4-related disease^12^. However, in our clinical observations, approximately one-third of patients (25/78) without evidence of AIP and PC showed a kind of diffused NSU FAPI uptake in the pancreas, which means the fibrotic or chronic inflammatory changes of the pancreatic tissue. The difference in SUVmax in the two groups (NSU vs. normal, 4.48 ± 0.98 vs. 2.01 ± 0.53, *P* < 0.05) was notable and can be identified by human eyes. Meantime, the difference of SUVmax for ^18^F-FDG PET/CT scan in two groups (NSU vs. normal, 2.23 ± 0.42 vs. 2.02 ± 0.44, *P* = 0.045), although statistically significant, was slight and was challenging to identify by human eyes. In the ROC analysis, SUVmax derived from ^18^F-FAPI PET/CT scan showed higher AUC, sensitivity, specificity, and accuracy in differentiating the pancreatic NSU than SUVmax derived from ^18^F-FDG (*P* < 0.05), and the Delong test of two ROC curves showed statistically different (Z = 5.348, *P* < 0.001). These results indicated that ^18^F-FAPI can detect chronic or fibrotic pancreas lesions that were negative in ^18^F-FDG.

In our cohort, the cutoff value of the normal pancreas was 3.25 for ^18^F-FAPI and 2.05 for ^18^F-FDG. Previous studies reported ^18^F-FAPI-SUVmax for the normal pancreatic tissues varied from 1.04 to 3.0^7,9^. Frederik L et al. et al. 7 conducted a head-to-head intra-individual comparison of biodistribution of ^68^Ga-FAPI and ^18^F-FDG PET/CT in cancer patients, and their results showed the pancreas uptake showed significantly lower for ^68^Ga-FAPI compared with ^18^F-FDG (1.82 vs. 1.99; *P* = 0.027). However, in our cohort, pancreas uptake showed no difference for ^18^F-FAPI compared with ^18^F-FDG (2.01 ± 0.53 vs 2.02 ± 0.44, *P* = 0.900). Cihan Gündoğan analyzed the ^68^Ga-FAPI-04 uptake in healthy tissues, and the pancreas showed a median SUVmax of 2.61 (minimal-maximal, 1.04 ~ 2.58)^13^, which is very close to our results.

In our cohort, heavy drinking, chronic liver diseases, and surgery of gastrectomy showed significant correlations with higher ^18^F-FAPI uptake. Previous study in a large-scale population shows that smoking, obesity, long-term drinking, diabetes, liver diseases, and long-term malnutrition are significantly associated with an increased risk of pancreatic disease^14^. Alcohol continues to be the an critical risk factor for chronic pancreatitis^15^. Therefore, alcoholism may have caused chronic pancreatitis. The surgery of gastrectomy, but not the intestine enterectomy, showed a correlation with pancreatic NSU, possibly due to the pancreatic fistula or biochemical leakage after gastrectomy^16^, which can lead to acute or chronic pancreatitis.

In our results, chronic liver disease, such as hepatitis or cirrhosis, but not liver metastasis, showed a correlation with pancreatic NSU. This can be explained. Hepatotropic viruses, such as hepatitis A, B, and E, were reported to affect of various extrahepatic tissues, such as the kidney, thyroid, pancreas, and bone marrow^17,18^. The presence of these viruses in pancreatic tissue induces fibrotic or chronic inflammatory changes resulting from the excessive deposition of the extracellular matrix, with the possibility of progression to metaplasia and subsequently, to malignant transformation^19^. Ying Kou also found the higher uptake of ^18^F-FAPI in the pancreas, with the SUVmax ± SD of (4.7 ± 1.5)^20^, which is very close to our results (SUVmax ^18^F-FAPI: 4.48 ± 0.98). However, they attributed this kind of higher uptake to physiological uptake. Whether the NSU is of clinical importance is not a done deal, ^18^F-FAPI may be a new way to visible the potential fibrous changes in the pancreas, which is not shown by the traditional imaging modalities.

In the laboratory test, patients in the NSU group showed a lower platelet count, higher mean platelet volume, and large platelet ratio for the complete blood cells analysis (*P* < 0.05), which means worse platelet morphology. Previous studies showed that the increase of atypical platelets may predict worse clinical outcomes^21,22^ and poor prognosis in patients with pancreatic cancer^23^. Also, patients in the NSU group showed higher levels of AST, total bilirubin, direct bilirubin, indirect bilirubin, and α-K-fucosidase in the serum (*P* < 0.05), which means impaired liver function or liver fibrosis in the patients with NSU of pancreas. In the logistic regression model, large platelet ratio, AST, and TBIL may be the independent factors for pancreatic ^18^F-FAPI uptake. This can be explained, as AST and the platelet are regarded as the important biomarkers in the evaluation of the liver fibrosis^24,25^.

To our knowledge, this is the first reported set of pancreatic NSUs for the ^18^F-FAPI. Whether there were NSU or not, the physician should carefully evaluate pancreatic uptake of ^18^F-FAPI, integrate the medical history and laboratory tests, and follow up for the final diagnosis in clinical practice.

Our study has certain limitations. The first limitation is the limited sample size. Second, there was no histo-pathological confirmation due to no clear or clinical indication of puncture for the NSU FAPI uptake. Thus, the follow-up is necessary, including the abdominal symptoms, laboratory test, or histo-pathological evidence if available.

In conclusion, we reported about 1/3 pancreatic NSU of ^18^F-FAPI, besides the AIP- or PC-related uptake. The NSU may be a result of a history of heavy drinking, chronic liver diseases, and surgery for gastrectomy. Patients with NSU showed lower levels of platelet count, larger platelet volume, and higher levels of AST, total bilirubin, direct bilirubin, indirect bilirubin, and α-L-fucosidase in the serum. Large platelet ratio, AST, and TBIL may be the independent factors for pancreatic ^18^F-FAPI uptake.

### Supplementary Information

Supplementary Table 1.

Supplementary Table 2.

## Data Availability

The datasets used and/or analysed during the current study available from the corresponding author on reasonable request.

## Abbreviations

AFP: Alpha-fetoprotein
AIP: Autoimmune pancreatitis
AIP: Autoimmune pancreatitis
ALT: Alanine aminotransferase
AST: Aspartate aminotransferase
AUC: Area under the curve
BMI: Body mass index
CA: Carbohydrate antigen
CAFs: Cancer-associated fibroblasts
CEA: Carcinoembryonic antigen
CRP: C reactive protein
CV: Coefficients of variation
CYFRA 19: Cytokeratin fragment 19
DBIL: Direct bilirubin
FAP: Fibroblast activation protein
FAPI: Fibroblast activation protein inhibitor
FDG: Fluorodeoxyglucose
IDBIL: Indirect bilirubin
IL-6: Interleukin 6
NLR: Neutrophil-to-lymphocyte ratio
NSE: Neuron-specific enolase
NSU: Non-specific uptake
PC: Pancreatic cancer
PET/CT: Positron emission tomography/computed tomography
ROC: Receiver operating characteristic
ROI: Region of interest
SCC: Squamous cell carcinoma
SD: Standard deviation
SUVmax: Maximum standardized uptake value
SUVmax: Maximum standardized uptake value
TBIL: Total bilirubin

## References

[1] Meyer C (2020). Radiation dosimetry and biodistribution of (68)Ga-FAPI-46 PET imaging in cancer patients. J. Nucl. Med..

[2] Sharma P, Singh SS, Gayana S (2021). Fibroblast activation protein inhibitor PET/CT: A promising molecular imaging tool. Clin. Nucl. Med..

[3] Çermik TF, Ergül N, Yilmaz B, Mercanoglu G (2022). Tumor imaging with Ga-DOTA-FAPI-04 PET/CT comparison With 18F-FDG PET/CT in 22 different cancer types. Clin. Nucl. Med..

[4] Dendl K (2021). FAP and FAPI-PET/CT in malignant and non-malignant diseases: A perfect symbiosis?. Cancers.

[5] Mori Y (2023). FAPI PET: Fibroblast activation protein inhibitor use in oncologic and nononcologic disease. Radiology.

[6] Dolensek J, Rupnik MS, Stozer A (2015). Structural similarities and differences between the human and the mouse pancreas. Islets.

[7] Giesel FL (2021). Head-to-head intra-individual comparison of biodistribution and tumor uptake of Ga-FAPI and F-FDG PET/CT in cancer patients. Eur. J. Nucl. Med. Mol. Imaging.

[8] Wei Y (2022). [(18)F]AlF-NOTA-FAPI-04: FAP-targeting specificity, biodistribution, and PET/CT imaging of various cancers. Eur. J. Nucl. Med. Mol. Imaging.

[9] Dendl K (2021). Ga-FAPI-PET/CT in patients with various gynecological malignancies. Eur. J. Nucl. Med. Mol. Imaging.

[10] Rohrich M (2021). Impact of Ga-68-FAPI PET/CT imaging on the therapeutic management of primary and recurrent pancreatic ductal adenocarcinomas. J. Nucl. Med..

[11] Luo Y (2021). Fibroblast activation protein-targeted PET/CT with (68)Ga-FAPI for imaging IgG4-related disease: Comparison to (18)F-FDG PET/CT. J. Nucl. Med..

[12] Zhang X, Song WY, Qin CX, Liu F, Lan XL (2021). Non-malignant findings of focal Ga-FAPI-04 uptake in pancreas. Eur. J. Nucl. Med. Mol. Imaging.

[13] Gundogan C, Guzel Y, Can C, Kaplan I, Komek H (2021). FAPI-04 uptake in healthy tissues of cancer patients in (68)Ga-FAPI-04 PET/CT imaging. Contrast Media Mol. Imaging..

[14] Alsamarrai A, Das SL, Windsor JA, Petrov MS (2014). Factors that affect risk for pancreatic disease in the general population: A systematic review and meta-analysis of prospective cohort studies. Clin. Gastroenterol. Hepatol..

[15] Yadav D, Lowenfels AB (2013). The epidemiology of pancreatitis and pancreatic cancer. Gastroenterology.

[16] Wu J (2022). Incidence and risk factors for postoperative pancreatic fistula in 2089 patients treated by radical gastrectomy: A prospective multicenter cohort study in China. Int. J. Surg..

[17] Jiang H, Li Y, Sheng Q, Dou X (2022). Relationship between Hepatitis B virus infection and platelet production and dysfunction. Platelets.

[18] Fiorino S, Cuppini A, Castellani G, Bacchi-Reggiani ML, Jovine E (2013). HBV- and HCV-related infections and risk of pancreatic cancer. JOP.

[19] Ferdek PE (2022). When healing turns into killing: The pathophysiology of pancreatic and hepatic fibrosis. J. Physiol..

[20] Kou Y (2022). Physiological tracer distribution and benign lesion incidental uptake of Al18F-NOTA-FAPI-04 on PET/CT imaging. Nucl. Med. Commun..

[21] Zheng YY, Wang L, Shi Q (2022). Mean platelet volume (MPV) and platelet distribution width (PDW) predict clinical outcome of acute ischemic stroke: A systematic review and meta-analysis. J. Clin. Neurosci..

[22] Akin H, Bilge O, Yavuz B, Ozkan S, Isik F (2022). The relationship between mean platelet volume and resistant hypertension. Clin. Exp. Hypertens..

[23] Yagyu T (2021). Decreased mean platelet volume predicts poor prognosis in patients with pancreatic cancer. BMC Surg..

[24] Hagström H, Talbäck M, Andreasson A, Walldius G, Hammar M (2020). Ability of non-invasive scoring systems to identify individuals in the population at risk for severe liver disease. Gastroenterology.

[25] Ginès P (2022). Population screening for liver fibrosis: Toward early diagnosis and intervention for chronic liver diseases. Hepatology.

