# Supplementary material for: Non-specific uptake of 18F-FAPI-04 in the pancreas and its related factors: a post-hoc analysis of an ongoing prospective clinical trial
Source: Sci Rep. 2024 May 15;14:11141. doi: 10.1038/s41598-024-62005-2 (PMC11096165; doi:10.1038/s41598-024-62005-2)
Supplement: Supplementary file 1 — Supplementary Table 1. [file 41598_2024_62005_MOESM1_ESM.docx]

**Supplementary table Primary malignancy or diagnosis of the included patients**

| **Primary malignancy or diagnosis** | **Cases** |
| --- | --- |
| Digestive system cancers |  |
| gastric cancer | 7 |
| liver cancer | 8 |
| colon cancer | 8 |
| rectal cancer | 3 |
| esophageal cancer | 5 |
| bile duct cancer | 6 |
| gastric stromal tumors | 3 |
| Lung cancer | 12 |
| Breast cancer | 1 |
| Reproductive system malignancies |  |
| ovarian cancer | 5 |
| uterine cancer | 5 |
| Urinary system tumors |  |
| bladder cancer | 3 |
| renal cancer | 1 |
| Head and neck cancers |  |
| maxillary sinus | 1 |
| thyroid | 1 |
| nasopharyngeal cancer | 2 |
| Hematological malignancies |  |
| multiple myeloma | 1 |
| lymphoma | 3 |
| Inflammatory diseases |  |
| Takayasu arteritis | 1 |
| connective tissue disease | 1 |
| tuberculosis | 2 |
| rheumatoid arthritis | 1 |
| In total | 80^a^ |

Note: a, two patients had two kind of primary tumors.
