# Supplementary material for: Non-specific uptake of 18F-FAPI-04 in the pancreas and its related factors: a post-hoc analysis of an ongoing prospective clinical trial
Source: Sci Rep. 2024 May 15;14:11141. doi: 10.1038/s41598-024-62005-2 (PMC11096165; doi:10.1038/s41598-024-62005-2)
Supplement: Supplementary file 2 — Supplementary Table 2. [file 41598_2024_62005_MOESM2_ESM.docx]

**Supplementary table The correlations between the SUVmax and blood cells, liver function indicators, tumor biomarkers and inflammatory indices**

| items | R | P | n |
| --- | --- | --- | --- |
| Blood test |  |  |  |
| Red cell count/10^12^*L^-1^ | 0.012 | 0.921 | 73 |
| Hemoglobin/ g*L^-1^ | -0.015 | 0.898 | 73 |
| Hematocrit/% | -0.001 | 0.993 | 73 |
| Mean erythrocyte volume/fL | 0.036 | 0.762 | 73 |
| hemoglobin/pg | 0.044 | 0.710 | 73 |
| hemoglobin concentration / g*L^-1^ | 0.013 | 0.915 | 73 |
| Erythrocyte width-CV/% | 0.190 | 0.104 | 73 |
| Erythrocyte width SD/fL | 0.111 | 0.348 | 73 |
| Platelet count/10^9^*L^-1^ | -0.296 | **0.011** | 73 |
| Platelet distribution width/fL | 0.061 | 0.610 | 73 |
| Mean platelet volume/fL | 0.223 | 0.058 | 73 |
| Large platelet ratio/% | 0.239 | **0.042** | 73 |
| Platelet accumulation/% | -0.252 | **0.031** | 73 |
| White cell count/10^9^*L^-1^ | 0.021 | 0.858 | 73 |
| Lymphocyte count/10^9^*L^-1^ | 0.098 | 0.408 | 73 |
| Monocyte count/10^9^*L^-1^ | 0.116 | 0.327 | 73 |
| Neutrophil count/10^9^*L^-1^ | -0.029 | 0.803 | 73 |
| Eosinophilic cell count/10^9^*L^-1^ | -0.212 | 0.070 | 73 |
| Basophilic cell count/10^9^*L^-1^ | 0.089 | 0.450 | 73 |
| Liver function |  |  |  |
| AST/U*L^-1^ | 0.256 | **0.030** | 72 |
| ALT/U*L^-1^ | 0.180 | 0.130 | 72 |
| AST/ALT | 0.120 | 0.315 | 72 |
| Alkaline phosphatase/U*L^-1^ | 0.149 | 0.211 | 72 |
| γ-glutamyl transpeptidase /U*L^-1^ | 0.148 | 0.216 | 72 |
| TBIL/μmol *L^-1^ | 0.310 | **0.009** | 72 |
| DBIL/μmol *L^-1^ | 0.306 | **0.010** | 72 |
| IDBIL/μmol *L^-1^ | 0.374 | **0.001** | 72 |
| Total protein/g*L^-1^ | 0.072 | 0.547 | 73 |
| albumin /g*L^-1^ | 0.116 | 0.330 | 73 |
| globulin/g*L^-1^ | 0.018 | 0.879 | 73 |
| albumin-globulin ratio/% | 0.022 | 0.865 | 73 |
| Total bile acids/μmol *L^-1^ | 0.180 | 0.242 | 44 |
| Blood glucose/mmol *L^-1^ | -0.052 | 0.671 | 69 |
| Tumor biomarkers |  |  |  |
| SCC/ ng*mL^-1^ | -0.043 | 0.790 | 41 |
| CEA/ ng*mL^-1^ | 0.173 | 0.187 | 60 |
| AFP/ ng*mL^-1^ | 0.009 | 0.955 | 39 |
| CA-125/U*mL^-1^ | 0.169 | 0.273 | 44 |
| CA-199/U*mL^-1^ | 0.170 | 0.225 | 53 |
| CA-724/U*mL^-1^ | 0.155 | 0.304 | 46 |
| CYFRA-19/ ng*mL^-1^ | 0.288 | 0.610 | 43 |
| NSE/ ng*mL^-1^ | -0.088 | 0.584 | 41 |
| Inflammatory indices |  |  |  |
| CRP/ mg*L^-1^ | -0.139 | 0.464 | 30 |
| IL-6/pg*mL^-1^ | 0.202 | 0.303 | 28 |
| IL-10/pg*mL^-1^ | 0.330 | 0.107 | 25 |
| IL-17/pg*mL^-1^ | 0.228 | 0.283 | 24 |

Abbreviations: CV, coefficients of variation; SD, standard deviation; AST, aspartate aminotransferase; ALT, alanine transaminase; TBIL, total bilirubin; DBIL, direct bilirubin; IDBIL, indirect bilirubin; CA, carbohydrate antigen; AFP, alpha-fetoprotein; CEA, carcinoembryonic antigen; SCC, squamous cell carcinoma antigen; NSE, neuron-specific enolase; CRP, C-reactive protein; IL, interleukin; CYFRA-19, cytokeratin fragment 19.
